# Astrocytic IL-6 Influences the Clinical Symptoms of EAE in Mice

**DOI:** 10.3390/brainsci6020015

**Published:** 2016-05-17

**Authors:** Maria Erta, Mercedes Giralt, Silvia Jiménez, Amalia Molinero, Gemma Comes, Juan Hidalgo

**Affiliations:** 1 Department of Cellular Biology, Physiology and Immunology, Animal Physiology Unit, Faculty of Biosciences, Universitat Autònoma de Barcelona, Bellaterra 08193, Spain; mariaerta@gmail.com (M.E.); merce.giralt@uab.es (M.G.); siljiba@gmail.com (S.J.); amalia.molinero@uab.es (A.M.); gemma.comes@uab.es (G.C.); 2 Institute of Neurosciences, Universitat Autònoma de Barcelona, Bellaterra 08193, Spain

**Keywords:** interleukin-6, astrocyte, EAE

## Abstract

Interleukin-6 (IL-6) is a multifunctional cytokine that not only plays major roles in the immune system, but also serves as a coordinator between the nervous and endocrine systems. IL-6 is produced in multiple cell types in the CNS, and in turn, many cells respond to it. It is therefore important to ascertain which cell type is the key responder to IL-6 during both physiological and pathological conditions. In order to test the role of astrocytic IL-6 in neuroinflammation, we studied an extensively-used animal model of multiple sclerosis, experimental autoimmune encephalomyelitis (EAE), in mice with an IL-6 deficiency in astrocytes (Ast-IL-6 KO). Results indicate that lack of astrocytic IL-6 did not cause major changes in EAE symptomatology. However, a delay in the onset of clinical signs was observed in Ast-IL-6 KO females, with fewer inflammatory infiltrates and decreased demyelination and some alterations in gliosis and vasogenesis, compared to floxed mice. These results suggest that astrocyte-secreted IL-6 has some roles in EAE pathogenesis, at least in females.

## 1. Introduction

Interleukin 6 (IL-6) is a multifunctional four-helix bundle cytokine, originally identified as a B-cell differentiation factor in 1985 [1], which has been linked to numerous biological functions. It is now known to be one of the main cytokines controlling the immune system, and in addition, it acts as a coordinator between the nervous and endocrine systems. IL-6 plays an essential role in the central nervous system (CNS) in many physiological, inflammatory and disease conditions, being able to exert dual actions (for a review, see [2]). Although many cells in the CNS produce IL-6 [3], astrocytes are the main producer [2,4].

Multiple sclerosis (MS) is one of the most common inflammatory disorders of the CNS and a leading cause of disability in young adults. It is estimated that 2–2.5 million people are currently living with this demyelinating disease worldwide [5]. Its pathological hallmarks consist of local demyelination, inflammation and variable axonal destruction. Experimental autoimmune encephalomyelitis (EAE) [6] is a well-known animal model to study this inflammatory condition. Cytokines are key mediators in the pathogenesis of inflammatory lesions in CNS, presenting both helpful and harmful effects [7]. IL-6 plays a crucial role in MS as demonstrated by its presence in acute and chronic active plaques of MS patients [8]. In addition, it has been shown that IL-6-deficient mice are resistant to EAE [9,10,11,12,13] presumably because of a lack of differentiation of naive T cells into MOG-specific T helper cells producing IL-17 (Th17) and the subsequent reduction of their infiltration into the CNS [14], although the exact mechanism is not fully understood.

A thorough understanding of the role of the cellular context in this and other diseases is necessary to clarify the putative roles of IL-6 in the CNS [15,16]. We recently characterized the role of astrocytic IL-6 in normal (basal) conditions by using transgenic mice with astrocyte-specific IL-6 deficiency, showing effects on behavior and body weight in a sex-dependent manner [17,18]. The role of astrocytic IL-6 during pathological situations remained to be assessed, and here, we report the initial studies with EAE.

## 2. Materials and Methods

### 2.1. Animals

The generation of astrocyte-IL-6 KO (Ast-IL6 KO) and floxed littermate mice, which served as controls, was as described previously [17]. A number of studies have shown that GFAP is primarily expressed in astrocytes of the CNS, with minimal expression in peripheral regions [19]. All mice were kept under constant temperature with free access to food (Harlan global diet 2918) and water. Ethical approval for the use and experimentation of all mice in this study was obtained from the Animal and Human Experimentation Ethics Committee of the Universitat Autònoma de Barcelona (nº 4017, approved 3 September 2015).

### 2.2. EAE Induction and Clinical Evaluation

For the induction of EAE, two-month-old male and female mice were used. EAE was induced by active immunization with MOG_35–55_ peptide. On Day 0, all mice, under isoflurane anesthesia, were injected subcutaneously into the hind flanks with an emulsion of 100 µL MOG_35–55_ (3 mg/mL) and 100 µL Complete Freund’s Adjuvant (CFA) (Sigma-Aldrich, St. Louis, MO, USA) supplemented with 4 mg/mL *Mycobacterium tuberculosis* H37RA (Difco, Detroit, MI, USA). In addition, animals received an intraperitoneal injection of 500 ng pertussis toxin (Sigma-Aldrich, St. Louis, MO, USA), which was repeated two days later.

After immunization, mice were examined daily, weighed and scored for EAE. The EAE clinical score was assessed for each animal according to the following criteria: 0 = no signs of disease, 0.5 = partial loss of tail tonus, 1 = loss of tail tonus, 2 = moderate hind limb paraparesis, 2.5 = severe hind limb paraparesis, 3 = partial hind limb paralysis, 3.5 = hind limb paralysis, 4 = hind limb paralysis plus partial front leg paralysis, 4.5 = moribund/total paralysis and 5 = death. Finally, for each animal, we determined the time to disease onset (clinical score ≥1), time to peak disease, peak-score, cumulative score (sum of all scores from disease onset to Day 20) and grade of remission (difference between peak score and outcome).

Three independent EAE experiments were carried out (Table 1). Experiment 1 was carried out for 0–22 days post-immunization (dpi); Experiment 2 was carried out for 0–20 dpi; and Experiment 3 was carried out for 0–46 dpi. For each experiment, littermates were used. Female mice from 20–22 dpi were grouped before comparison to 46 dpi mice. Male Ast-IL-6 KO mice did not differ from male floxed mice at 20–22 dpi and, thus, were not examined at 46 dpi. In all cases, all surviving mice were euthanized at the indicated days post-immunization by decapitation. Spinal cords were immediately removed and fixed for 48 h in 4% paraformaldehyde and embedded in paraffin for immunohistochemistry (IHC) and histochemistry (HC) analyses. Spinal cords from additional healthy female mice were processed in parallel.

### 2.3. IHC and HC Analysis

Embedded paraffin tissues were cut into 8 μm-wide sections in a microtome (Leica, Germany) and mounted in Superfrost slides (Thermo Scientific, Waltham, MA, USA).

Microglia were identified by lectin HC (tomato lectin from *Lycopersicon esculentum*, Sigma-Aldrich 1:500 in Tris-buffered saline (TBS) with 0.5% triton X-100 (TBS-t)). Astrocytes were identified by GFAP IHC (rabbit anti-Glial Fibrillary Acidic Protein from DakoCytomation Denmark, 1:1200 in blocking buffer). Lymphocytes were identified by CD3 IHC (rabbit anti-human CD3, Dako A0452, 1:100 in blocking buffer). Spinal cord sections were also stained with hematoxylin-eosin and with Luxol Fast Blue solution (LFB) (0.1%, overnight al 37 °C) and counterstained with Cresyl violet (0.1%, 1 min), to assess the number of cellular infiltrates and demyelination in grey matter, respectively.

Sections for IHC and HC were preincubated for 1 h with the blocking buffer (0.5% BSA in TBS-t) and then incubated with the primary antibodies (GFAP, CD3) or tomato lectin overnight at 4 °C, followed by 1 h at room temperature (RT) (GFAP, CD3) or at 37 °C (lectin). For CD3 IHC, a previous antigen retrieval step was performed with protease type XIV (Sigma P5147). After washing in TBS, sections were incubated with either horseradish peroxidase-coupled streptavidin (Vector Labs, Burlingame, CA, USA, 1:600, 1 h) for HC or biotinylated secondary antibody (Vector Labs, Burlingame, CA, USA, 1:300, 1 h at RT) followed by washes and horseradish peroxidase-coupled streptavidin (Vector Labs, Burlingame, CA, USA, 1:600, 1 h) for IHC. The immunoreactivity was visualized by using 0.033% H_2_O_2_ in 0.5 mg/mL 3,3-diaminobenzidine-tetrahydrochloride (DAB) in Tris buffer (TB) for 4–30 min at room temperature. Reaction was stopped with TB, washed, dehydrated and mounted in DPX (distyrene-plasticiser-xylene) (Sigma, St Louis, MO, USA). Images at 100× (GFAP) or 200× (lectin, CD3) were taken in a bright field Nikon Eclipse 90i microscope (Nikon Instruments Europe BV, Amsterdam, The Netherlands) and acquired with a Nikon digital camera DXm1200F (Nikon Instruments Europe BV, Amsterdam, The Netherlands) and Nikon Act-1 version 2.70 software (Nikon Instruments Europe BV, Amsterdam, The Netherlands) from different brain areas and spinal cord. Finally, to quantify staining areas, intensity and the number of cells, images were analyzed using ImageJ software (NIH, Bethesda, MD, USA). Histological analyses were performed on at least two sections per mouse. Control sections for non-specific binding analysis (where primary antibody or tomato lectin was not used) were included routinely.

For GFAP IHC, the percentage of stained area (at 100×) was measured in different regions of the spinal cord from EAE-induced mice. Six to twelve images per animal were examined. White and grey matter areas of spinal cords were also analyzed (12 images per animal and area). A threshold was set for each area to better define cells from tissue background. Because tomato lectin HC stains both microglia and vessels, the quantification of staining using ImageJ was supplemented with manual counts of microglial cells showing a basal (ramified), reactive or fully-activated (round) morphology and of the number of vessels; these were assessed in the same regions as for GFAP IHC analysis, at 200×. In addition, total numbers of microglia/macrophage infiltration areas in spinal cord were recorded. For CD3 IHC, the percentage of stained area was measured in the spinal cord (10 images per area and animal). In LFB/Cresyl violet staining, total numbers of cellular infiltrates in grey matter of spinal cord were counted, and afterwards, a color deconvolution plugin from ImageJ software was used in order to separate colors from LFB and Cresyl violet to be able to quantify the demyelination by analyzing the percentage of LFB-stained area in spinal cord. The threshold set gave 100% of area of the spinal cord covered by LFB staining for healthy female mice. To standardize, the same threshold was used for female and male mice. Demyelination and a decrease of the area covered by LFB caused by EAE were readily detected in both male and female mice.

### 2.4. Statistical Analysis

Values in text and figures are shown as the mean ± standard error of the mean (SEM). Statistical analyses were performed using SPSS statistical software (version 17.0 for Windows, SPSS Inc., Chicago, IL, USA). *p* ≤ 0.05 was considered significant in all analyses.

For clinical evaluations and body weight changes, we used the general linear model (GzLM) for repeated measures, the generalized estimated equations test (GEE), with genotype (floxed *vs.* Ast-IL-6 KO mice) and sex as the main factors. The *post-hoc* Student *t*-test or the Mann–Whitney *U*-test was used to identify significant differences between Ast-IL-6 KO and floxed animals at specific time periods. For the remaining variables analyzed, two-way ANOVA (with genotype and sex as the main factors) or GzLM was used for each sex separately followed by *post-hoc* tests when appropriate.

## 3. Results

### 3.1. Lack of Astrocytic IL-6 Alters the Clinical Course of EAE in Ast-IL-6 KO Mice and Ameliorates EAE Symptomatology in a Sex-Dependent Manner

**Figure 1:**
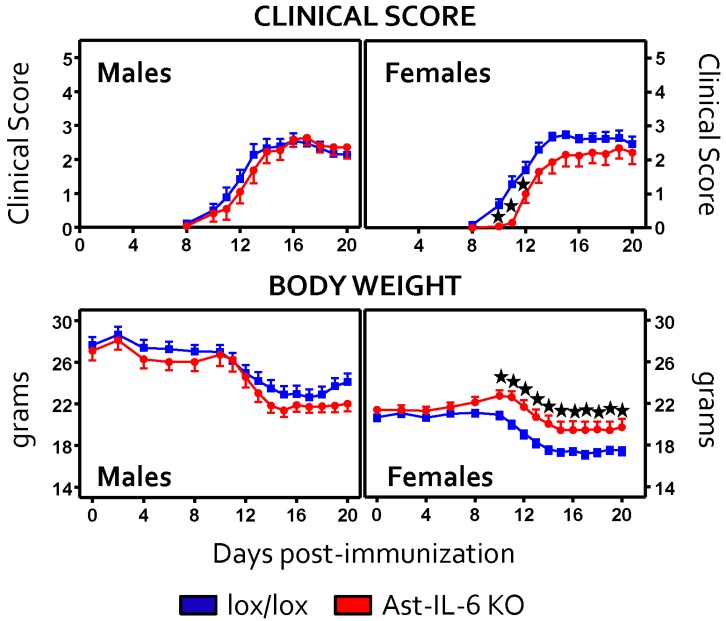
Clinical course of EAE, in both floxed (*n* = 18 males, 28 females) and Ast-IL-6 KO (*n* = 11 males, 23 females) mice up to 20 dpi. Results are the mean ± SEM of data pooled from Experiments 1–3 (Days 0–20 dpi; Table 1). ★ *p* at least <0.05 *versus* floxed mice at specific days following a *post-hoc* analysis.

Both genotypes showed the prototypical ascending paralysis course with body weight loss (Figure 1). The clinical score and the body weight changes observed following MOG_35–55_ immunization up to 20 dpi in the Ast-IL6 KO and floxed mice are shown in Figure 1. Ast-IL6 KO females showed a significantly reduced clinical score and increased body weight with respect to female floxed mice, a genotypic difference that was not observed in the male mice. Average incidence of the disease ranged from 90% to 100%, and there were no significant differences in mortality rate (Table 2).

When the day of disease onset was analyzed using sex and genotype as the main factors, a significant genotypic difference was observed for both sexes (Table 2). In contrast, the peak score, cumulative score and grade of remission at 20 dpi were not different between genotypes for either sex (Table 3). These results suggest that while the disease is delayed and the severity is somewhat lower at the early stages in female mice, it is not affected by astrocytic IL-6 deficiency later on.

### 3.2. Reduced Cellular Infiltrates and Demyelination in the Spinal Cord of Ast-IL-6 KO Female Mice

**Figure 2:**
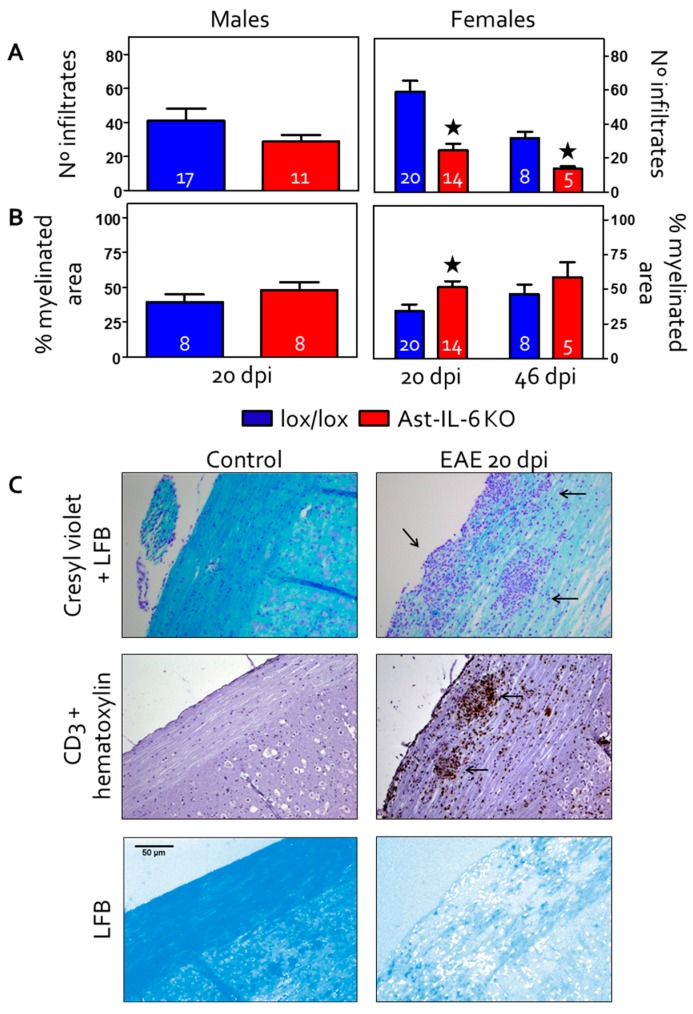
Assessment of the total number of cellular infiltrates (**A**) and demyelination (**B**) in the longitudinal lumbar-cervical spinal cord white matter from EAE-induced animals at 20 dpi (for both females and males Ast-IL6 KO and floxed mice) and 46 dpi (females). Number of mice per group as indicated. ^★^ *p* < 0.05 between Ast-IL6 KO and floxed mice. (**C**) Representative sections from female mice showing infiltrates (arrows) in spinal cord stained with Cresyl violet (top) and CD3 (middle) in non-immunized (control) and immunized (20 dpi) floxed mice. The bottom panel shows the demyelination of the spinal cord as revealed by Luxol Fast Blue (LFB) staining following color deconvolution. All images at magnification 100×.

The number of inflammatory infiltrates in the longitudinal lumbar-cervical spinal cord of control and EAE-induced animals was assessed in females and males at 20–22 dpi (for simplicity, data from 20–22 dpi were grouped and are referred to as 20 dpi). Females were also assessed at 46 dpi (Figure 2A,C). The total number of infiltrates in white matter was counted in a Cresyl violet/Luxol Fast Blue staining and in a CD3 IHC counterstained with hematoxylin; a mean value of both stainings was then calculated for each animal and used for statistical analysis. As shown in Figure 2A, there is a significant decrease in the number of infiltrates in Ast-IL6 KO females at both 20–22 and 46 dpi compared to floxed female mice. Ast-IL-6 KO males did not show a significant difference compared to floxed males (Figure 2A).

Demyelination in spinal cord white matter was assessed measuring the percentage of Luxol Fast Blue-stained area (Figure 2B,C). Healthy animals (0 dpi) had 100% area covered by LFB staining. Following EAE, Ast-IL-6 KO females showed a significantly lower Luxol Fast Blue-stained area, indicating greater demyelination, compared to floxed female mice at 20 dpi, but not at 46 dpi (Figure 2B). Ast-IL-6 KO males did not show a significant difference in Luxol Fast Blue-stained area compared to floxed males (Figure 2B).

### 3.3. Reduced Gliosis and Vasogenesis in Spinal Cord and Brain of EAE-Induced Ast-IL-6 KO Females

**Figure 3:**
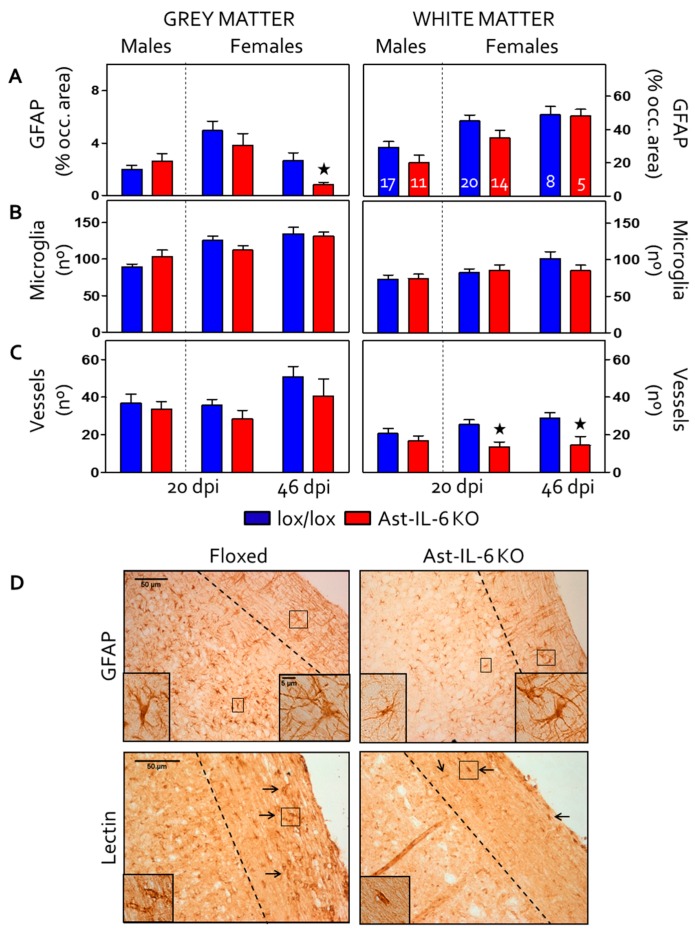
(**A**–**C**) Results from GFAP and lectin stainings in the spinal cord (grey and white matter) of EAE-induced animals at 20 dpi (for both females and males Ast-IL6 KO and floxed mice) and 46 dpi (females). Number of mice per group as indicated. GFAP overall immunostaining (**A**) and the number of lectin-positive microglia (**B**) and vessels (**C**) are shown. ^★^ *p* < 0.05 *vs.* floxed mice. (**D**) Representative GFAP at 100× (top) and lectin at 150× (bottom) stainings of 46 and 20 dpi females, respectively. Arrows indicate vessels. The discontinuous line separates grey matter (left) from white matter (right). All inserts are at 400×; at the top they, show astrocytic morphology in both grey and white matter, and at the bottom, they show a vessel.

Gliosis was evaluated by GFAP (astrocytes) and lectin (microglia) staining in the spinal cord of the EAE-induced mice (Figure 3A,B,D). The area occupied by GFAP immunostaining was measured in both the grey and white matter (Figure 3A). A significant decrease in GFAP immunostaining in grey matter was observed in Ast-IL-6 KO female mice compared to floxed female mice at 46 dpi, accompanied by a less reactive morphology of astrocytes (Figure 3D, top, left inset). No genotypic difference in GFAP immunostaining was observed for females or males in grey matter at 20 dpi or in the white matter for females and males at the times studied.

Regarding microgliosis in spinal cord (Figure 3D, bottom), quantification of the occupied area did not show significant differences between genotypes (data not shown). As quantification of lectin staining is not able to separate microglia from blood vessels, the number of microglia was counted. No significant genotypic difference in the number of microglia was observed for either females or males in both the grey and white matter of the spinal cord at the days studied (Figure 3B). These results indicated that astrocytic IL-6 deficiency did not impact microgliosis in either the grey or white matter of the spinal cord.

Finally, vasogenesis was analyzed by counting lectin-stained vessels in the spinal cord of EAE-induced mice (Figure 3D, bottom, arrows and insets). In female mice, the number of vessels in the white matter was significantly reduced in Ast-IL-6 KO mice compared to floxed mice at both 20–22 and 46 dpi (Figure 3C). No genotypic difference was observed for the number of vessels in the white matter for males or for the number of vessels in the grey matter for females and males at the times studied.

## 4. Discussion

IL-6 is implicated in the pathogenesis of autoimmune disorders, such as MS in humans [20,21]. A critical role of IL-6 in the animal model of MS, EAE, has been demonstrated by a number of studies: systemic IL-6 KO mice are resistant to EAE [9,10,11,12,13], and neutralization of IL-6 with antibodies leads to a reduced disease [22], by not well-defined mechanisms. Moreover, studies have demonstrated that the transgenic expression of IL-6 in the CNS by viral systems reduces EAE [23] and that the systemic administration of IL-6 also reduces the clinical symptoms in a viral model of EAE [24]. Thus, IL-6 can potentiate, but also inhibit EAE, reflecting the complexity of its actions. Key questions remain, however, including the identity of the key cell types that produce and respond to IL-6 and whether the critical actions of IL-6 are peripheral or central.

Because the production of IL-6 during the course of EAE arises from diverse cellular sources both in the periphery and in the CNS, the specific contribution of each source of IL-6 to the development of the disease needs to be established. We have previously demonstrated a role of CNS IL-6 in regulating EAE, because mice expressing IL-6 only by astrocytes (GFAP-IL6-IL-6 KO mice) were capable of developing the atypical EAE known to occur in GFAP-IL6 mice [13]. Furthermore, in adoptive transfer experiments, EAE is less severe in IL-6KO mice than in wild-type mice, which suggests that IL-6 locally mediates the disease in the CNS [10]. Thus, although EAE has always been considered a disease mostly induced peripherally, it seems that CNS IL-6 may also play an important role. Because astrocytic IL-6 plays a major role in neuroinflammation [25,26] and because astrocytes are the most abundant cell in the CNS, astrocytic IL-6 seemed to be an excellent candidate to examine as the key regulator of EAE in the CNS.

We have previously shown that compared to floxed mice, Ast-IL-6 KO mice exhibit a number of altered behaviors under normal (basal) conditions, including changes in activity, anxiety and learning; a prosurvival role of astrocytic IL-6 is also apparent [17,18]. Here, we present results from studies involving a neuropathological condition, MOG_35–55_-induced EAE. In contrast to results in systemic IL-6 KO mice, astrocytic-specific IL-6 deficiency is unable to prevent typical signs of EAE induction and has no prominent neuropathologic effects. However, astrocyte IL-6 KO mice did show significant delays of the onset of the EAE, at least in female mice, ameliorating the clinical signs in the early stages of EAE.

Autoreactive T-cells can result in inflammatory demyelination of the CNS, and knowing that the frequency of Tregs in MS patients is unchanged from controls [27] (although their function is impaired) could be a clue to the decreased demyelination seen only in Ast-IL-6 KO females, the only group that presented a decrease in T lymphocyte infiltration. Thus, a lower EAE score at early stages could be due to an initial impaired T cell infiltration of the CNS. This possibility is consistent with our results showing that Ast-IL-6 KO female (but not male) mice had a decreased number of infiltrates in the spinal cord and lower scores for clinical signs. However, it is important to note that we only carried out Cresyl violet and CD3 immunostaining for T cells, so we cannot rule out that a change in T cell subpopulations is responsible for the different clinical signs observed in Ast-IL-6 KO female mice. IL-6 has a major role in Th17 cell differentiation from naive CD4^+^ T-cells (reviewed in [28]), particularly in the EAE model [14,29]. EAE-resistant IL-6 KO mice show a deficiency in Th17 cell infiltrates in the CNS [30]. When responsiveness to IL-6 is experimentally eliminated in T helper cells, mice show resistance to EAE induction, as the IL-21 pathway is intact, but not active in the absence of IL-6 signaling [31]. Th17 cells produce IL-17 (among other cytokines), which enhances IL-6 production by astrocytes, which, in turn, induces the differentiation of Th17 cells in a positive feed-back loop between IL-17 and IL-6 via activation of NF-*κ*B and STAT-3 [32,33]. This loop may be compromised in our Ast-IL-6 KO animals, where astrocyte production of IL-6 has been eliminated. However, since Ast-IL-6 KO mice only show a delay in EAE, but are not resistant to EAE, we can speculate that this astrocytic loop is not necessary for the development of the disease; and/or that neuronal, endothelial and microglial IL-6 could instead allow this positive feedback between IL-17 and IL-6. In order to test these hypotheses, a detailed study of the exact lymphocytic population present in the infiltrates would be important, particularly with further backcrossing to C57/Bl6 mice.

Somewhat surprisingly, considering the importance of astrocytic IL-6 in neuroinflammation [2,4,25,26], we did not observe dramatic effects of astrocytic IL-6 deficiency on either astrocytes or microglia at the times studied. This result makes it unlikely that changes in glial cell reactivity are underlying the differences in clinical signs between Ast-IL-6 KO mice and floxed mice. Nevertheless, overall staining and cell morphologies are limited approaches, and more detailed studies are needed to assess other roles of glial cells (other than IL-6 production by astrocytes) in clinical signs of EAE. Finally, regarding the number of vessels, we observed a decrease in Ast-IL-6 KO mice, which is in agreement with results in mice overexpressing IL-6, which show extensive revascularization, both in basal conditions [25,26] and after an injury [34], indicative of a role of IL-6 in vasogenesis. Other studies also support the idea that IL-6 promotes vasogenesis [35]. Probably, this is secondary to the number and/or type of inflammatory cells present in the spinal cord, since no differences were observed in male mice.

The extent of the reduction of IL-6 in astrocytes of the Ast-IL-6 KO mice *in vivo* has yet to be determined. However, studies of cultured astrocytes from the Ast-IL-6 KO mice demonstrated that the astrocytes are deficient in IL-6 production. In these studies, analysis of culture supernatant after 24 h of stimulation (10 ng/mL of LPS and 10 ng/mL of INF-γ) showed that astrocytes from floxed mice produced approximately 13 ng/mL of IL-6, whereas astrocytes from Ast-IL-6 KO mice only produced 2 ng/mL IL-6 [17,18]. Regardless of the extent of the astrocytic IL-6 deficiency in the Ast-IL-6 KO mice, a delay in onset to clinical symptoms was evident in females, including less demyelination at 20–22 dpi in accordance with the lower clinical scores. However, this was a transient effect, and sometime after 20–22 dpi, the EAE was similar in both genotypes (as indicated by the lack of significant differences in demyelination at 46 dpi), indicating the astrocytic IL-6 no longer played a role.

## 5. Conclusions

In conclusion, we have shown that lack of astrocytic IL-6 is not sufficient to prevent EAE disease, but it is able to delay the disease and to ameliorate clinical scoring and the inflammatory milieu in female mice. These results support the idea that the local CNS production of IL-6 is important in this disease. Several interesting questions remain to be addressed. For example, due to the delayed onset of clinical signs of disease in Ast-IL-6 KO females, it will be important to analyze in detail the priming and inflammatory infiltrates in the CNS of female and male Ast-IL-6 KO mice and their floxed controls. Furthermore, studies of males at later dpi may reveal genotypic differences at later stages. Moreover, a comparison of the immunized Ast-IL-6 KO and floxed mice with a CFA immunized control group could reveal specific EAE effects in Ast-ILK-6 KO mice. Future studies will address these and other relevant issues relative to the role of astrocytic IL-6 in the EAE.

## Figures and Tables

**Table 1:** Number of mice per genotype, sex and experiment.

Genotype	Experiment 1 (0–22 dpi)		Experiment 2 (0–20 dpi)		Experiment 3 (0–46 dpi)
	Males	Females	Males	Females	Females
Floxed	7	3	11	17	8
Ast-IL-6 KO	3	7	8	8	8

dpi, days post-immunization; Ast, astrocyte.

**Table 2:** EAE disease.

EAE	Females		Males	
	Ast-IL-6 KO	Floxed	Ast-IL-6 KO	Floxed
Incidence	21/23	28/28	11/11	17/18
Mortality	3/23	0/28	0/11	0/18
Day of onset	13.38 ± 0.59 *	11.57 ± 0.29	12.27 ± 0.52 *	11.65 ± 0.49

Results shown are pooled data from Experiments 1–3 separated by sex and genotype. For each animal, time to disease onset was defined by a clinical score ≥1. For statistical analysis, a two-way ANOVA for genotype (floxed *vs.* Ast-IL-6 KO mice) and sex as the main factors was performed. * *p* < 0.05 *vs.* floxed mice.

**Table 3:** EAE clinical course.

Clinical Course	Females (0–20 dpi)		Females (46 dpi)		Males (0–20 dpi)	
	Ast-IL-6 KO (*n* = 15)	Floxed (*n* = 20)	Ast-IL-6 KO (*n* = 8)	Floxed (*n* = 8)	Ast-IL-6 KO (*n* = 11)	Floxed (*n* = 18)
Time of peak score	14.30 ± 0.53	14.80 ± 0.49	22.5 ± 2.46 *	15.5 ± 1.92	15.18 ± 0.80	13.76 ± 0.6
Peak score	2.80 ± 0.23	3.45 ± 0.16	3.68 ± 0.50	3.18 ± 0.23	3.18 ± 0.18	3.14 ± 0.12
Cumulative score	19.40 ± 2.55	24.16 ± 1.32	88.87 ± 21.84	72.18 ± 10.35	20.32 ± 1.64	22.61 ± 1.34
Grade of Remission	0.64 ± 0.13	0.91 ± 0.11	1.00 ± 0.34	1.43 ± 0.27	0.82 ± 0.26	20.32 ± 1.64

Results shown are pooled data from Experiments 1–3 separated by sex and genotype. For simplicity, data from 20–22 dpi were grouped and are referred to as 20 dpi. For each animal, we determined time to peak disease, peak-score, cumulative score (sum of all scores from disease onset to Days 20 and 22 combined or 46), and the grade of remission (difference between peak score and outcome). Results are the mean ± SEM. For statistical analysis at 0–20 dpi, a two-way ANOVA for genotype (floxed *vs.* Ast-IL-6 KO mice) and sex as the main factors was performed. For the females at 46 dpi, a one-way ANOVA for genotype (floxed *vs.* Ast-IL-6 KO mice) was performed. * *p* < 0.05 *vs.* floxed mice.
